# Cryopreservation of *Hydractinia symbiolongicarpus* Sperm to Support Community-Based Repository Development for Preservation of Genetic Resources

**DOI:** 10.3390/ani12192537

**Published:** 2022-09-22

**Authors:** Aidan L. Huene, Jack C. Koch, Lucía Arregui, Yue Liu, Matthew L. Nicotra, Virginia M. Weis, Terrence R. Tiersch

**Affiliations:** 1 Department of Surgery, Thomas E. Starzl Transplantation Institute, University of Pittsburgh, Pittsburgh, PA 15213, USA; 2 Pittsburgh Center for Evolutionary Biology and Medicine, University of Pittsburgh, Pittsburgh, PA 15213, USA; 3 Aquatic Germplasm and Genetic Resources Center, School of Renewable Natural Resources, Louisiana State University Agricultural Center, Baton Rouge, LA 70820, USA; 4 Department of Immunology, University of Pittsburgh, Pittsburgh, PA 15213, USA; 5 Department of Integrative Biology, Oregon State University, Corvallis, OR 97331, USA

**Keywords:** *Hydractinia*, sperm cryopreservation, cnidaria, germplasm repository, open hardware, 3-D printing, fertilization, larvae

## Abstract

**Simple Summary:**

*Hydractinia symbiolongicarpus* is an emerging model organism in which cutting-edge genomic tools and resources are being developed for use in a growing number of research fields. One limitation of this model system is the lack of long-term storage for genetic resources. In this study, approaches for *Hydractinia* sperm cryopreservation were established for the first time. Open hardware and 3-D printing were used to facilitate animal husbandry, sperm handling, and cryopreservation. *Hydractinia* sperm at a concentration of 2 × 10^7^ cells/mL stored at 4 °C for as long as 6 d were able to achieve 50% fertilization rate. A fertilization rate of 41–69% was observed using sperm equilibrated with 5, 10, or 15% (*v*/*v*) cryoprotectant (dimethyl sulfoxide or methanol) for 20 min, cooled at a rate of 5, 10, or 20 °C/min from 4 °C to −80 °C, at a cell concentration of 1 × 10^8^ sperm/mL, in 0.25 mL French straws. Establishing repository capabilities for the *Hydractinia* research community will be essential for future development, maintenance, protection, and distribution of genetic resources. More broadly, these generalizable approaches can be used as a model to develop germplasm repositories for other cnidarian species.

**Abstract:**

*Hydractinia symbiolongicarpus* is an emerging model organism in which cutting-edge genomic tools and resources are being developed for use in a growing number of research fields. One limitation of this model system is the lack of long-term storage for genetic resources. The goal of this study was to establish a generalizable cryopreservation approach for *Hydractinia* that would support future repository development for other cnidarian species. Specific objectives were to: (1) characterize basic parameters related to sperm quality; (2) develop a generalizable approach for sperm collection; (3) assess the feasibility of in vitro fertilization (IVF) with sperm after refrigerated storage; (4) assess the feasibility of IVF with sperm cryopreserved with various sperm concentrations; (5) evaluate feasibility of cryopreservation with various freezing conditions, and (6) explore the feasibility of cryopreservation by use of a 3-D printed open-hardware (CryoKit) device. Animal husbandry and sperm collection were facilitated by use of 3-D printed open hardware. *Hydractinia* sperm at a concentration of 2 × 10^7^ cells/mL stored at 4 °C for 6 d were able to achieve 50% fertilization rate. It appeared that relatively higher sperm concentration (>5 × 10^7^ cells/mL) for cryopreservation could promote fertilization. A fertilization rate of 41–69% was observed using sperm equilibrated with 5, 10, or 15% (*v*/*v*) cryoprotectant (dimethyl sulfoxide or methanol) for 20 min, cooled at a rate of 5, 10, or 20 °C/min from 4 °C to −80 °C, at a cell concentration of 10^8^/mL, in 0.25 mL French straws. Samples cryopreserved with the CryoKit produced a fertilization rate of 72–82%. Establishing repository capabilities for the *Hydractinia* research community will be essential for future development, maintenance, protection, and distribution of genetic resources. More broadly, these generalizable approaches can be used as a model to develop germplasm repositories for other cnidarian species.

## 1. Introduction

*Hydractinia symbiolongicarpus* is a colonial cnidarian and an established model for evolutionary developmental biology, stem cell biology, regeneration, and allorecognition [1,2,3]. In recent years, efforts to improve *Hydractinia* as a model system have included generation of laboratory strains for use by the research community, sequencing of these strains through the Hydractinia Genome Project (https://research.nhgri.nih.gov/hydractinia, accessed on 31 July 2022), and establishment of methods to produce transgenic animals via the random integration of exogenous DNA [4] or targeted integration via CRISPR/Cas9-mediated gene knock-in [5].

An increasing limitation to the expanded use of *Hydractinia* as a model is the lack of long-term storage options for genetic resources. Over the years, laboratories have collected and bred hundreds of genotypically distinct colonies, while simultaneously generating strains bearing various transgenes. In all cases, these animals have had to be maintained as live animals or they would be lost. While this is possible because *Hydractinia* colonies can be maintained for decades under laboratory conditions, it is increasingly costly in terms of labor and space [6]. These costs are often minimized by reducing colonies to the smallest possible size, and only expanding them via clonal reproduction when needed for experiments. These colonies remain vulnerable to accidents, disease, and improper handling, which can result in death and permanent loss of genotypes important to previous, current, and future research.

Germplasm cryopreservation is an efficient tool to support preservation and management of valuable genetic resources [7]. As an immediate benefit, cryopreservation would allow for a much-needed “back-up” of animals that are of considerable current research value. As a long-term benefit beyond laboratory use, cryopreserved stocks would allow user groups from across the research community to store and access samples on demand rather than requiring time and resources to grow or collect new animals [8]. While the ultimate goal would be cryopreservation of germplasm and somatic tissues from all life stages, here we focused on *Hydractinia* sperm as the most amenable to cryopreservation based on previous success in corals [9] and the anemone *Nematostella* (Matt Gibson and Shane Merryman, personal communication).

Successful cryopreservation of sperm requires the balance of multiple parameters [10]. These include the storage temperature and time that elapses between sperm collection and freezing, sperm concentration at the time of freezing, choice and concentration of cryoprotectant, cooling method and rate, thawing method and rate, and the conditions under which thawed sperm will be used for fertilization [11]. In addition, to facilitate adoption of the cryopreservation approaches by the user community, the feasibility of use of standardized hardware with low cost that can be easily accessed needs to be investigated. Thus, the goal of this study was to establish a generalizable cryopreservation approach for *Hydractinia* that would support future repository development for other cnidarian species. Specific objectives were to: (1) characterize basic parameters related to sperm quality; (2) develop a generalizable approach for sperm collection; (3) assess the feasibility of in vitro fertilization (IVF) with sperm after refrigerated storage; (4) assess the feasibility of IVF with sperm cryopreserved with various sperm concentrations; (5) evaluate feasibility of cryopreservation with various freezing conditions, and (6) explore the feasibility of cryopreservation by use of a 3-D printed open-hardware (CryoKit) device. In this study, approaches for sperm cryopreservation for *Hydractinia* were established for the first time. This generalizable approach can be used as a model to develop germplasm repositories for other cnidarian species.

## 2. Materials and Methods

### 2.1. Ethics and Animal Care

Animal care was overseen by separate Institutional Animal Care and Use Committees at the University of Pittsburgh and the Louisiana State University Agricultural Center. *Hydractinia symbiolongicarpus* is a marine invertebrate lacking a central nervous system and is not regulated by specialized guidelines. All animals used in this study were maintained in continuous culture as detailed below.

Experimental work was performed at the Aquatic Germplasm and Genetic Resources Center (AGGRC) in Baton Rouge (LA, USA) with animals transported in 50-mL tubes by overnight shipping from the University of Pittsburgh (Pittsburgh, PA, USA). Colonies were maintained and grown as previously described [5] and cultured for at least 2 wk before use in experiments. Briefly, colonies (Figure S1) were established on 25 × 75 mm glass microscope slides and cultured in 38 L (10 gal) glass aquarium tanks using artificial seawater (ASW) (Instant Ocean Reef Crystals, Spectrum Brands, Blacksburg, VA, USA) at 29–31 ppt, held at 22–23 °C with an 8 h:16 h (light:dark) photoperiod. Adult colonies were fed 4-day-old *Artemia* nauplii on Monday, Wednesday, and Friday. On Tuesday and Thursday, colonies were fed a suspension of puréed oysters (fresh caught, shucked, puréed, aliquoted, flash frozen in liquid nitrogen, and stored at −20 °C).

In this study, two half siblings were crossed, including a male (colony 291-10) and a female (colony 295-8). After 16 h in the dark, male and female colonies were exposed to light and moved into separate containers filled with ASW. Gametes naturally released approximately 1–1.5 h after light exposure.

### 2.2. Sperm Characterization

*Hydractinia* are dioecious and have gonozooids (reproductive polyps) that bear multiple gonophores (gamete-filled structures) that release either sperm or eggs. Healthy *Hydractinia* release gametes daily. After fertilization, embryos develop into planula larvae (1–4 days) before permanently attaching to surfaces and metamorphosing into juvenile primary polyps. Sperm were released in white “clouds” or “streams” and were collected by use of a Pasteur pipette or micropipette. For IVF, 20–30 sperm streams were collected from 10 male slides. Samples (1–3 mL) were transferred to a 50-mL conical tube, and adjusted to a final volume of 15 mL with addition of filtered seawater (FSW, artificial seawater filtered through 0.45-μm polyethersulfone) that was prepared by use of a Rapid-Flow Sterile Disposable Bottle Top Filters (Thermo Scientific Nalgene, Waltham, MA, USA, catalog #295-4545).

To count sperm concentration, an aliquot of sperm was combined with a dilution (between 1:1 and 1:10) of 30% glycerol in FSW to stop motility. The sample was loaded onto a Makler^®^ counting chamber (SEFI Medical Instruments Ltd., Irvine Scientific, Santa Ana, CA, USA), and viewed with phase-contrast illumination at 200× magnification (Olympus CX41RF, Tokyo, Japan). The sample concentration was counted twice according to an established protocol [12], and the average concentration was expressed as cells/mL.

Sperm were motile in seawater, and thus did not require activation. Motility was quantified using a computer-assisted sperm analysis (CASA) system (CEROS model; Hamilton Thorne, Inc., Beverly, MA, USA) with phase contrast and 200× magnification. The settings used were based on a previous study [13]. Briefly, motility and VCL (curvilinear velocity) were recorded within 10 µL samples loaded in the Makler chamber^®^. Cell detection was set at a minimum of 25 pixels for contrast and 6 pixels for cell size. In each measurement, 100 frames were captured at a rate of 60 frames/s. Sperm with an average of >20 μm/s measured path velocity (VAP) were counted by the program as being progressively motile.

### 2.3. Development of Standardized Sperm Collection Approaches

To facilitate sperm handling, a sperm collection chamber was designed by use of free computer-aided design (CAD) software (Tinkercad, version 4.7, Autodesk, San Rafael, CA, USA). The design was exported as a stereolithography (STL) file and imported into a 3-D printer slicer software (Simplify3D, version 4.0, Cincinnati, OH, USA) to control the printing process (Table S1). Collection chambers were printed using polylactic acid (PLA) filament (ZYLtech Engineering, Spring, TX, USA) on a Prusa i3 MK3 3-D printer (Prusa Research, Prague, Czech Republic) (Table S2).

### 2.4. In vitro Fertilization with Sperm after Refrigerated Storage

Short-term storage of sperm samples is important for cryopreservation research and processing, because it allows researchers to perform various experiments. Preliminary trials indicated that sperm motility declined rapidly at room temperature (22 °C), and no progressive motility (only twitching) could be observed by 7 h. In contrast, sperm kept at 4 °C retained progressive motility for 7 h and non-progressive twitching for 23 h. As such, keeping sperm at 4 °C (refrigerated) was applied to evaluate feasibility of short-term storage. Approximately 150 clouds of sperm were collected and stored in a 50-mL conical tube at 4 °C for 6 d. On each subsequent day following initial sperm collection, 3 mL of refrigerated sample with a total of 2 × 10^7^ sperm (6.7 × 10^6^ cells/mL) were used to fertilize 100–300 eggs in a total volume of 30 mL FSW. In addition, fresh sperm were collected daily to fertilize eggs as a control. This feasibility experiment was used to inform subsequent experiments, and thus, no biological replicates were performed.

### 2.5. In vitro Fertilization with Sperm Cryopreserved at Various Concentrations

Adjustment of sperm concentration is critical in cryopreservation for aquatic animals [14]. Sperm were collected into 50-mL conical tubes and centrifuged for 20 min at 1500× *g*, which resulted in a visible white pellet. The supernatant was removed, and the pellets were resuspended in FSW and adjusted to the appropriate stock concentration (2 × 10^9^, 1 × 10^9^, 2 × 10^8^, 1 × 10^8^, or 2 × 10^7^ cell/mL). Sperm were mixed with an equal volume of 10% DMSO (resulting in final concentrations of sperm of 1 × 10^9^, 5 × 10^8^, 1 × 10^8^, 5 × 10^7^, and 1 × 10^7^ cells/mL in 5% DMSO), loaded into 0.25-mL French straws (IMV Technologies, L’Aigle, France) during 20 min of equilibration (from initial mixing with cryoprotectant to start of the freezing program). After equilibration, samples were cooled at 20 °C/min from 4 to −80 °C and stored in liquid nitrogen for 21 h. After thawing, a 10-μL sample was collected for assessment of sperm concentration and the remaining volume (~0.24 mL) used for IVF. About 100–280 eggs were used for IVF with each sperm sample. This feasibility experiment was also used to inform subsequent experiments, and thus, no biological replicates were performed.

### 2.6. Cryopreservation with Various Cryopreservation Conditions

Nine slides of males were placed in the 3-D printed sperm collection chamber filled to the top with ASW (~80 mL). Sperm were collected, centrifuged at 1560× *g* for 30 min at 20 °C, resuspended with FSW to 2 × 10^8^ cells/mL, and stored at 4 °C until they were prepared for freezing (~3 h).

To evaluate the effects of various cryopreservation conditions, sperm were mixed with an equal volume of 10%, 20%, or 30% of DMSO or methanol in FSW (final concentrations of 5%, 10%, and 15% for each cryoprotectant), loaded into 0.25-mL French straws (IMV Technologies), and held at 4 °C in a controlled-rate freezer (Minitube of America, Verona, WI, USA, IceCube 14M, SY-LAB) during 20 min of equilibration. Equilibrated samples were cooled to −80 °C with three different cooling rates: 5, 10, and 20 °C/min. Frozen samples were held at −80 °C for at least 5 min before transfer and storage in liquid nitrogen.

### 2.7. Cryopreservation with 3-D Printed Open Hardware

Based on the previous experiment, a cryopreservation protocol was chosen to assess the feasibility of 3-D printed CryoKit [15]. Sperm were collected following the procedure described in Section 2.6, and sperm concentration was evaluated and adjusted following the procedure described in Section 2.2 to a final concentration of 2 × 10^8^ cells/mL. To prepare for freezing, diluted sperm were mixed with an equal volume of DMSO or methanol in FSW (to final concentrations of 5% and 10% for each cryoprotectant and 1 × 10^8^ sperm/mL), loaded into 0.25-mL French straws, and held at 20 °C for 20 min equilibration. A type-T thermocouple (Omega Engineering Inc., Norwalk, CT, USA) was inserted in a straw filled with FSW to record the cooling rate at 1 s by use of a HOBO 4-channel thermocouple logger (Onset Computer Corporation, Bourne, MA, USA). Equilibrated samples were cooled to −80 °C at 16.5 °C/min (CryoKit configuration 1B: no legs, open float). The cooling rate was calculated from 20 to −80 °C. Frozen samples were held below −80 °C for at least 5 min before transfer and storage in liquid nitrogen.

### 2.8. Thawing and IVF

Based on feasibility trials for various sperm concentration (2.5), IVF procedures for investigation of cryopreservation conditions (2.6) and open hardware (2.7) were revised for improvement of efficiency and standardization. After at least 48 h of storage and within 30 min of the expected spawning time, straws were removed from liquid nitrogen and immediately plunged into FSW at room temperature (22 °C) for 10 s. A 25-μL aliquot from each thawed sperm sample was pipetted into one well of a 12-well plate and diluted with ~0.5 mL FSW to cover the bottom of the well. Sperm were used for fertilization within 20 min after thawing.

**Figure 1:**
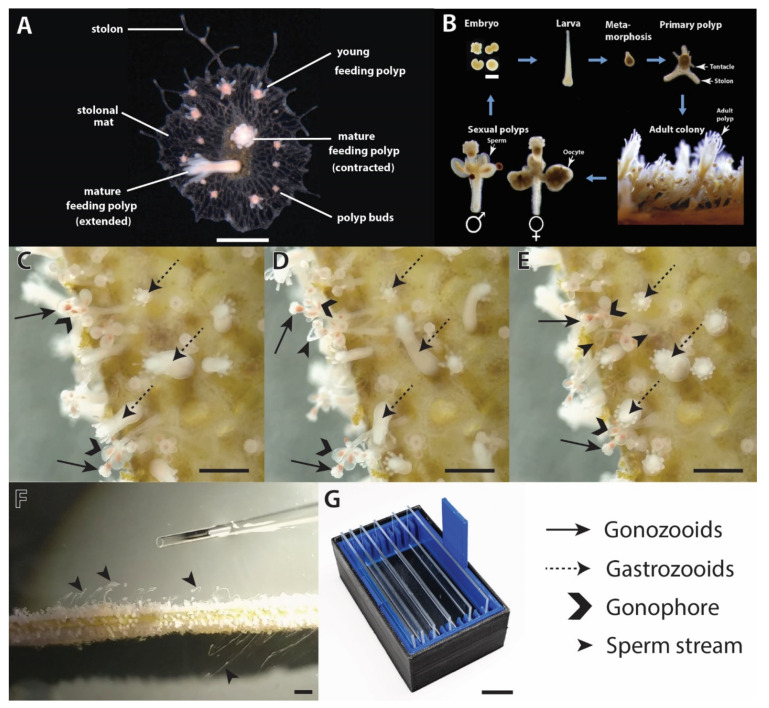
*Hydractinia* colony morphology, life history, and sperm release and collection. (**A**) Major morphological structures of a colony (scale bar = 1 mm). (**B**) Life cycle (scale bar = 200 µm). (**C**) Polyps directly prior to sperm release (scale bar = 2 mm). (**D**) Polyps during sperm release (scale bar = 2 mm). (**E**) Polyps during late stages of sperm release and polyp retraction (scale bar = 2 mm). (**F**) Collection of sperm streams by use of pipet (scale bar = 2 mm). (**G**) The 3-D printed *Hydractinia* sperm collection chamber (black) with the slide rack (blue) inserted with slides (scale bar = 1.9 cm). Panels (**A**,**B**) are adapted from [4,16] and licensed under CC BY 4.0 (link: https://creativecommons.org/licenses/by/4.0, accessed on 31 July 2022).

To prepare for IVF, 10 female slides were placed in a small plastic bin with approximately 0.5 L of ASW; eggs were released ~1.5 h after light exposure and were collected by straining the water through a 20-μm cell strainer. For the preliminary trials, eggs were added to 15 mL of the appropriate sperm suspension, and the volume was adjusted to 30 mL with FSW and transferred to a 100-mm polystyrene Petri dish. For evaluation of cryopreservation conditions and the CryoKit, eggs were added to the 12-well plates in ~1 mL of FSW. Due to the variability in egg release, eggs were distributed as evenly as possible among the wells based on that day’s release (between 20 and 934). The number of eggs used was counted manually with a dissection microscope within 10 min of combining gametes. If counting could not be completed within 10 min, an image was taken of the eggs through transmitted light with a diffuser using the camera app (v. 12.0.01.76) of a Samsung Galaxy S22 mobile phone (Suwon, Korea). Within 1 h, embryos (Figure 1A,B) began to cleave. Within 24 h, the embryos had developed into larvae. The number of larvae were counted between 24–48 h after fertilization to determine fertilization rate that was expressed as: (total larvae/total eggs) × 100%.

## 3. Results

### 3.1. Sperm Characterization

Motilities from 35 sperm streams were individually characterized with the CASA system. Sperm were motile in ASW at 29–31 ppt, and thus, no additional activation was needed. Curvilinear velocity (VCL) for each sperm stream was 50.8 ± 26.2 μm/s (ranging from 5% to 95%), percent motility was 37 ± 22% (ranging from 10% to 100%), and concentration was 9 ± 5 × 10^6^ sperm/mL (ranging from 1.0 × 10^6^ to 1.7 × 10^7^ sperm/mL).

### 3.2. Standardized Sperm Collection

Sperm were released in “clouds” or “streams” from individual gonophores (Figure 1C–E) and were collected using a Pasteur pipette or micropipette (Figure 1F) to prepare a relatively concentrated initial sperm suspension. However, collecting individual sperm streams is inefficient (time-consuming), and requires training and labor. As such, a *Hydractinia* sperm collection chamber (Figure 1G) was designed with an integrated slide rack. This chamber (www.thingiverse.com/thing:3661286 (accessed on 31 July 2022)) could incubate as many as ten slides bearing male colonies in a compact space (with ~80 mL of water), thus eliminating the need to collect individual sperm streams with pipettes. This enabled collection of ~10^9^ sperm per day (a 100-fold increase compared with pipetting).

### 3.3. In vitro Fertilization with Sperm after Refrigeration Storage

**Figure 2:**
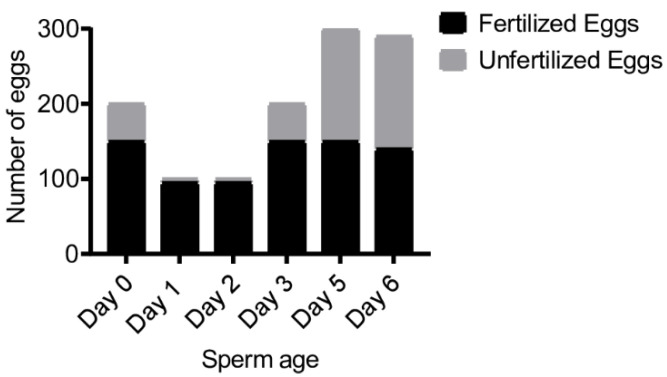
Sperm fertilization capability after short-term storage at 4 °C for as long as 6 d. Each day, 2 × 10^7^ sperm cells from the same collection aliquot were used to fertilize freshly collected eggs in 30 mL of FSW. On days 1 and 2, only ~100 eggs were available for IVF. On the other days, a surplus of eggs was collected.

In this preliminary trial, >95% eggs were fertilized daily in the control group (fresh sperm collected daily), indicating that there were no appreciable differences in egg quality for fertilization. On day 0, a total of 2 × 10^7^ sperm (3 mL) were mixed with approximately 200 eggs and resulted in 150 embryos. Because 50 eggs remained unfertilized, it was estimated that 2 × 10^7^ sperm were capable of fertilizing ~150 eggs. On each subsequent day, the same amount of refrigerated sperm was mixed with as many eggs as we could collect. Twenty million sperm (2 × 10^7^) could consistently fertilize ~150 eggs after 3, 5, and 6 d of refrigerated storage at 4 °C (Figure 2). On days 1 and 2, only ~95 eggs could be collected due to spawning variation, nearly all of which were fertilized. All embryos developed and metamorphosed into normal juvenile colonies. This trial indicated that 2 × 10^7^ sperm could consistently fertilize up to ~150 eggs within 6 d of refrigerated storage.

### 3.4. In vitro Fertilization with Sperm Cryopreserved at Various Concentrations

**Figure 3:**
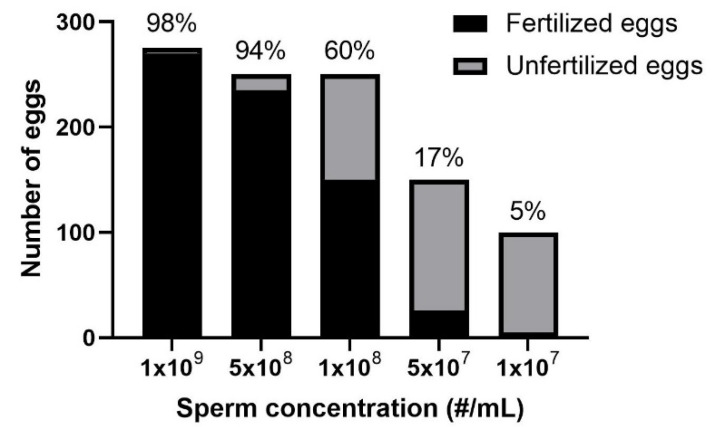
In vitro fertilization with frozen sperm at different concentrations. Each thawed sperm sample was exposed to a different number of eggs. In each case, the number of eggs collected was manually estimated to be a surplus of what each respective sperm sample could fertilize based on the concentration. Percentage fertilization is indicated on top of the bars.

The previous experiment indicated a relationship between fertilization rate and the ratio of sperm to eggs, and thus, the feasibility of fertilization with sperm cryopreserved at various concentrations was explored. The concentration of each sample after thawing was comparable to those before freezing. After thawing (Figure 3), sperm frozen at 10^9^ and 5 × 10^8^ sperm/mL were able to fertilize >95% eggs. Reduction of sperm number appeared to decrease fertilization rate, from 95% (with 5 × 10^8^ sperm/mL) to 5% (with 1 × 10^7^ sperm/mL). All embryos developed into larvae and were able to metamorphose into a primary polyp with no visual abnormalities.

### 3.5. Cryopreservation with the Controlled-Rate Freezer Using Various Cryopreservation Conditions

**Figure 4:**
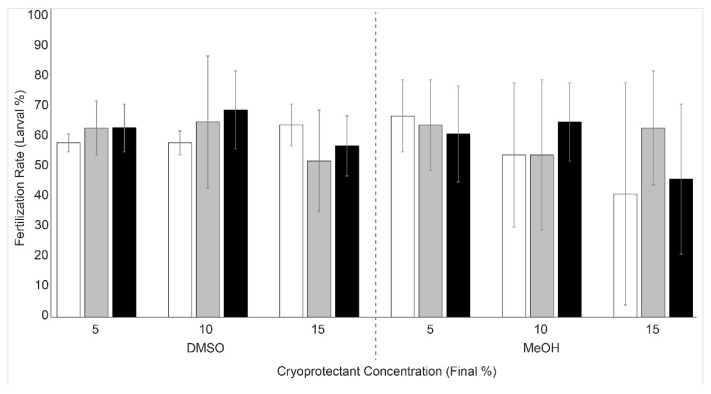
Fertilization rate of *Hydractinia* by use of sperm cryopreserved with different cryoprotectants and cooling rates (*n* = 3). Fertilization rate is reported as mean (±SD) percentage calculated by dividing the number of larvae by the initial number of eggs. White bars represent cooling at 5 °C/min, grey bars represent cooling at 10 °C/min cooling, and black bars represent cooling at 15 °C/min. DMSO: dimethyl sulfoxide; MeOH: methanol.

Sperm cryopreserved in various types of cryoprotectants (5, 10, and 15% DMSO or methanol) and different cooling rates (5, 10, and 20 °C/min) were able to fertilize *Hydractinia* eggs (Figure 4, Table S3) with a fertilization rate of 41–69%. The fertilization rate was >50% in all treatments except for samples treated with 15% methanol at 5 and 20 °C/min. Samples treated with DMSO and 5 °C/min showed relatively lower variation (3–7% SD) than other treatment groups (8–37%).

### 3.6. Cryopreservation with 3-D Printed Open Hardware

**Figure 5:**
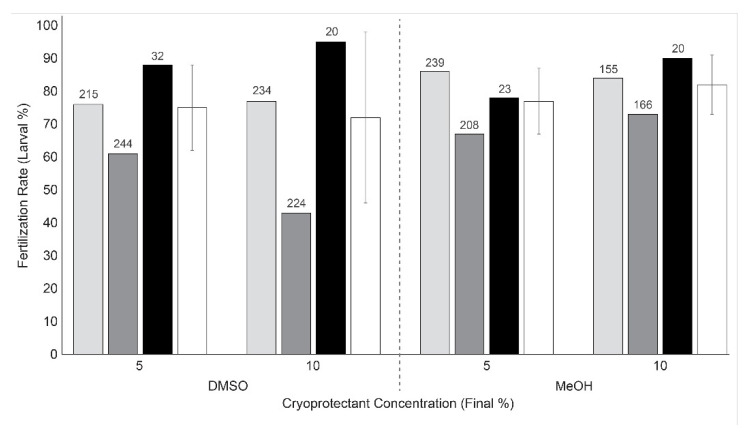
Fertilization rate of *Hydractinia* eggs by use of sperm cryopreserved using the CryoKit with various cryoprotectant conditions (*n* = 3). Fertilization rate is reported as larval percentage calculated by dividing the number of larvae by the initial number of eggs. Light grey bars represent replicate 1, dark grey bars represent replicate 2, black bars represent replicate 3, and white bars represent the mean (±SD) of the three replicate trials. Numbers above the bars indicate the initial number of eggs counted. DMSO: dimethyl sulfoxide; MeOH: methanol.

Sperm cryopreserved with the 3-D printed open hardware (CryoKit) were used to fertilize between 155 and 244 eggs for replicates 1 and 2, and between 20 and 32 eggs in Replicate 3 (Figure 5, Table S4). The cooling rate of samples processed by the CryoKit was 16.5 °C/min. Four of the conditions (DMSO and methanol at 5 and 10%) produced thawed sperm that could fertilize *Hydractinia* eggs, resulting in an average fertilization rate of 72–82%.

## 4. Discussion

The rapid proliferation of new lines and mutants in most aquatic model species requires them to be maintained as live animals, which is expensive and risky without cryopreservation as a storage method. This pilot study serves as a feasibility investigation in the management of *Hydractinia* genetic resources through sperm cryopreservation and germplasm repositories. With this first step, the *Hydractinia* research community can further develop reproducible and robust cryopreservation techniques. As a model for cnidarian species, investigation in *Hydractinia* cryopreservation can provide insights for a consistent and foundational approach toward cryopreservation of other cnidarians for the ultimate purpose of repository development and establishment of repository networks. By having this long-term goal in mind, we can more systematically work toward developing, protecting, maintaining, distributing, and utilizing an expanding pool of cnidarian genetic resources.

### 4.1. Sperm Characterization

This study provides insight into the basic characteristics of *Hydractinia* sperm that have not been previously observed. Considerable variation in motility (spanning from 10 to 100%) and concentration (spanning from 1.0 × 10^6^ to 1.7 × 10^7^ sperm/mL) among sperm streams reflects the variability encountered when traditionally collecting individual streams with pipetting. These findings reinforce the need to standardize collection methods and sperm concentrations. Future studies can also address other outstanding questions related to these characteristics. For example, when and how are sperm activated? A better understanding of how these features could affect cryopreservation, especially among different genotypes, would be useful in expanding and making protocols more robust for *Hydractinia* and potentially other cnidarian species.

### 4.2. Standardized Sperm Characterization Approach

The sperm collection approach developed herein provides expanded opportunities for standardized sample processing and quality evaluation. Recent advances in consumer-level fabrication technologies (e.g., 3-D printing) enable custom design of open hardware to support community-level efforts for repository development [17,18]. In this study, customizing the 3-D printed collection chamber greatly increased the efficiency in sperm collection. The previous collection method via Pasteur pipette or micropipette was labor-intensive and posed logistical problems if more than one person was collecting. Given our previous approach, collecting all sperm would be possible but would require filtration of all the water from the bin (~2 L) or having access to a centrifuge with a relatively large capacity (e.g., 5 L). Thus, by customizing a chamber to minimize the collection volume (<80 mL) and maximize the total number of sperm (as many as ten slides bearing clonal *Hydractinia* in a single chamber), the processing efficiency and standardization capability were greatly improved.

### 4.3. Refrigerated Storage

Refrigerated storage of sperm prior to use or freezing provides flexible operation timing and shipping of germplasm for processing. *Hydractinia* sperm stored at 4 °C for as long as 6 d could fertilize the numbers of eggs that were comparable to those fertilized by freshly collected sperm. This result suggested that it is possible to store sperm in FSW at 4 °C even longer (>6 d) and still produce viable embryos. Identifying these basic storage conditions is useful in cases when resources are not available to process on-site, and samples must be transported to another facility for processing and storage. Future studies should compare fertility across a range of storage temperatures with longer storage times when appropriate and integrate that with freezing experiments to evaluate the effects of storage on cryopreserved sample survival. In addition, extender solutions can influence the quality and retention of fertility of sperm during storage [10,19,20]. Future studies should also address these solutions as a means to extend the fertilization window for eggs. Although mixing of gametes ≤30 min after release has been the community guideline for producing quality embryos in *Hydractinia*, and may be the case in corals as well [21], it has not been established quantitatively, and it is possible that egg storage at cool temperatures may extend fertility (and add flexibility in scheduling).

### 4.4. Sperm Concentration in Cryopreservation

Before cryopreservation, adjustment of sperm concentration is critical for success for aquatic species [14,22]. Our results showed a positive relationship to sperm concentration, with fertilization increasing from 5% to 98% by increasing the sperm concentration. This is likely to be related to the ratio of sperm and eggs, which was determined to be ~2.5 × 10^6^ cryopreserved sperm to 150 eggs. This can be translated to fertilization of ~1000 eggs with a single 0.25 mL straw of frozen sperm at a concentration of 1 × 10^8^/mL. This experiment was performed as a range-finding and feasibility trial (and thus no replicates were applied). Future studies can further investigate the minimum and maximum fertilization ratios based on findings in the present study.

### 4.5. Cryopreservation Conditions

Freezing at 1 × 10^8^ sperm/mL with 5, 10, and 15% of DMSO or methanol at the three cooling rates tested (5, 10 or 20 °C/min) resulted in variable post-thaw fertilization. In some cases, there was a high variability among replicates, ranging from 3 to 90% fertilization with standard deviations ranging from 3 to 37%. This variation could be caused by factors such as handling procedures (e.g., time and temperature for each step), and male conditioning (temperature and diet variation), which should be further investigated. The most obvious variable in these IVF experiments was the number of eggs that were exposed to the same dose of sperm. Variation in spawning output as well as manual estimation and distribution of eggs is not precise. However, in all conditions, thawed sperm were capable of fertilizing fresh eggs. Some of the most consistent fertilization results at each freezing rate were for samples cryopreserved with 10% DMSO or methanol at 20 °C/min, 10% methanol at 10 °C/min, 5% DMSO or methanol at 10 °C/min, and 15% DMSO or 5% methanol at 5 °C/min. These observations show that there generally is no single optimum for cryopreservation, and various combinations of treatments and conditions can produce acceptable results. Future experiments and efforts to store germplasm by the user community can use these results to guide development of suitable cryopreservation conditions. In addition, the effects of cryopreservation on genetic makeup for subsequent generations can be further investigated.

While there are no other *Hydractinia* cryopreservation protocols to directly compare to, there are protocols that have been developed for sperm from various coral species [9,23,24]. With regard to cooling rates, there are several differences that make these studies difficult to compare. First, the equilibration time and temperature used were different [24,25] or not explicitly quantified [26]. Second, the ending temperatures used to calculate the freezing curve were different, where one study used −80 °C [24], but the other two used the coldest achievable temperature (between −110 and −130 °C). Theoretically, the ending temperature should not affect the rate calculation if the freezing rate is constant, but unless the temperature is monitored while the samples are being frozen, fluctuations are difficult to account for. Thus, differences in procedures such as these make direct comparisons of studies difficult, and it is critical that all details surrounding the freezing process be documented to ensure reproducibility in results and sample quality [11]. For this reason, only two of the studies can be referenced for reproducibility and generally compared in relation to their cooling rate [24,25]. Both studies used an equilibration temperature between 24–29 °C and equilibration time of 15 [25] or 20 min [24], where in this study, equilibration was at 4 °C for 20 min. The selection of 4 °C as the equilibration temperature in our study was in part due to the use of a controlled-rate freezer, although samples frozen using the CryoKit were equilibrated at room temperature (~22 °C).

One of the coral protocols cryopreserved 1-mL samples in 2-mL cryovials [24], whereas the other two studies [25,26] cryopreserved samples in 0.25-mL French straws. French straws offer several advantages over traditional cryovials. The straws require less storage space and can be easily processed manually in the case of a few samples, or more efficiently in high-throughput with automated filling, labeling, and sealing for hundreds to thousands of samples. In addition, samples can generally be cooled in French straws at a faster rate than in cryovials, in large part due to their higher surface area-to-volume ratio (which can also decrease variability during freezing). In cryovials, there is potentially more variation across the sample volume as material on the periphery could freeze more rapidly than that closer to the center. In addition, vials typically have thicker walls with greater insulative potential, slowing heat removal from the sample.

Similar results to those in the present study were found in coral species, with DMSO and methanol used as cryoprotectants, yielding a fertilization rate of 45–50% [9,23,24]. A notable effort is being made in gamete cryopreservation for conservation of coral species and their symbionts due to importance of corals to reef biodiversity and their overall decline in health and prevalence globally over the past several decades [27,28,29,30]. Coral cryopreservation is also made challenging because of the limited time frame for sample collection (e.g., some species only spawn once per year) [31]. The daily spawning of *Hydractinia* in laboratory conditions can provide an opportunity to use them as a model to study germplasm cryopreservation for other cnidarian species. Other topics that could be investigated in future work include whether offspring produced from cryopreserved sperm mature into full adults and whether male-to-female ratios are affected.

### 4.6. Cryopreservation with 3-D Printed Open Hardware

Incorporating the CryoKit into our approach was important to developing a protocol that is generalizable and easily accessible to the user community. The CryoKit method, and others like it, can be less precise than using controlled-rate freezers. While programmable freezers offer the advantage of being standardized, they are expensive (e.g., >USD 25,000 for entry level freezers) for most researchers and facilities. Developing portable and more affordable tools that perform reproducible cooling rates will accelerate availability of cryopreservation throughout the cnidarian community. In this study, similar cooling rates and fertilization results were obtained by use of the 3-D printed open-hardware CryoKit, compared to using the controlled-rate programmable freezer. 

The PLA filament used for 3-D printing does not become as brittle or stiff as other plastics when exposed to cryogenic temperatures [32] making 3-D printed objects safe and useful for such applications [33,34,35]. Various devices can typically be fabricated at low cost (e.g., <USD 5 for the CryoKit used herein) using consumer-level printers (e.g., USD 250 or less) that offer high resolution, flexibility, and accessibility. There are large internet-driven user communities for these printers, and thousands of videos (such as on YouTube) are available for printer setup, training, and troubleshooting. In addition, design files can be shared on a number of sites (e.g., Thingiverse, NIH 3D Print Exchange, and Github) for sharing and distribution. In this way, devices used in cryopreservation and repository development can be developed, shared, and standardized within research communities, greatly reducing costs of cryopreservation, and making reliable methods widely available [36]. However, distributed production systems such as this must be accompanied by quality control (QC) and quality assurance (QA) programs to ensure that samples meet minimum thresholds for repository use [37,38].

### 4.7. Approaches to Repository Development for Aquatic Species

Overall, the success in the present study of using a generalizable approach for *Hydractinia* sperm provides further evidence that cryopreservation protocols need not be considered as being species specific. For example, a single generalized protocol was applied to more than 20 species within the genus *Xiphophorus* and two other species in the genus *Poecilia* (a genera of live-bearing fish) to enable repository development to safeguard the genetic resources of these valuable biomedical model species [12]. The present study offers evidence that substantial repository-level benefits can be realized by generalizing cryopreservation at the application level, rather than trying to optimize new protocols on a species-by-species basis, or step-by step level [11]. Based on the frequency and predictability of gamete release, *Hydractinia* could be a useful model for the development of repositories for corals. When properly fed, *Hydractinia* colonies will release gametes on a daily basis approximately 1 h after exposure to light. Many coral species release gametes only once or twice per year, and the factors that trigger spawning are not well understood [31,39,40]. Using *Hydractinia* could help narrow the conditions and treatments to be tested in a targeted coral species. In addition, *Hydractinia* propagates quickly and reaches sexual maturity in only a few months.

This study was focused on the application of a pathway that can be directly scaled for use with hundreds of animals and multiple laboratories. Work addressing repository development in previous studies, with blue catfish (*Ictalurus furcatus*) for example [41,42], can be generalized to *Hydractinia* because the approaches used are the same. This includes the use of French straws that can be filled, sealed, and labeled using automated equipment (e.g., the Minitube Quattro system at the AGGRC can process 15,000 straws per hour). In addition, cryopreservation in *Hydractinia* can be directly transferred from a central facility, such as the AGGRC (aggrc.com, accessed on 31 July 2022), to on-site work within an existing laboratory by use of high-throughput mobile cryopreservation capabilities [43], or by establishment of full high-throughput cryopreservation capabilities such as the creation of a central *Hydractinia* Stock Center (for economic analysis, see [44]). Development of in-house cryopreservation capabilities within research laboratories will be greatly strengthened by the recent developments in 3-D printing described above (e.g., [15]) including fabrication of customized probes for monitoring and storing temperature information [45], and the potential for sharing of open hardware design files for production of inexpensive, reproducible freezing devices that can be integrated with strong quality management programs (e.g., [37,38]). 

A centralized stock center and germplasm repository is a necessity for well-developed research organisms, and the success of centralized repositories can be driven by collaboration among laboratories and the sharing of tools, systems, and resources throughout the communities. For example, mouse resources are largely centralized with The Jackson Laboratory (https://www.jax.org, accessed on 31 July 2022); zebrafish databases (https://www.zfin.org, accessed on 31 July 2022) and lines are found within the Zebrafish International Resource Center (ZIRC, University of Oregon, Eugene, OR, USA); *Drosophila* utilizes the Bloomington *Drosophila* Stock Center (BDSC, Indiana University Bloomington, Bloomington, IN, USA); *Caenorhabditis elegans* and other worm-related models localize their resources in WormBase (https://www.wormbase.org, accessed on 31 July 2022); and *Xenopus* related resources are found in Xenbase (https://www.xenbase.org, accessed on 31 July 2022). Having a wealth of such resources and information available for these communities makes these model systems much more useful and available to investigators, whereas model systems that require development of basic tools including cryopreservation can be more challenging on many levels.

## 5. Conclusions

This study showed that it is possible and a worthwhile endeavor to pursue *Hydractinia* sperm cryopreservation as a long-term storage option for genetic resources. Specifically, we demonstrated that sperm cooled at 5, 10, or 20 °C/min in 5%, 10%, or 15% DMSO or methanol at a concentration of 10^8^ cells/mL in 0.25 mL French straws were able to fertilize eggs, which developed into larvae. While all conditions tested resulted in fertilization, the cryopreservation conditions that produced the most consistent results were the 5% DMSO at a cooling rate between 10–20 °C/min. These samples exhibited the most consistent results between the controlled-rate freezer and the CryoKit and would also minimize potential cytotoxicity effects of higher cryoprotectant concentrations (e.g., 15% DMSO) and lengthen exposure to cryoprotectant at slower freezing rates (e.g., 5 °C/min). In our experience, a population of juvenile colonies typically contains sufficient numbers to establish a strain for propagation by asexual reproduction (i.e., they will grow into healthy adults) or by breeding to produce subsequent generations. While sperm cryopreservation is a giant stride forward in the *Hydractinia* community, future studies should explore cryopreservation of adult tissues, which would preserve the existing adult lines and would also serve as an indirect stock for germplasm. Cryopreserving multiple tissue types would also greatly enhance the utility of *Hydractinia* as a model system for cnidarian genetics. In addition, this work can also provide a guide to researchers seeking to develop cryopreservation approaches in other cnidarian species.

Future studies should establish a standardized approach for the storage, shipment, and use of frozen *Hydractinia* samples that can be made available throughout the research community. Current models for this would include development of repositories or a repository system, and the potential incorporation of these entities into a community-based stock center. An existing model for such organization exists in ZIRC, which maintains more than 43,000 research lines of zebrafish as frozen sperm. In addition, to assist standardization of protocols and approaches, it may be useful to establish community-level mechanisms to design and share inexpensive devices that can be used to support users across a wide range of experience and skill levels in culture, spawning, and cryopreservation of *Hydractinia*. Lastly, cryopreservation and repository development should be expanded to include additional germplasm and somatic cell types.

## Data Availability

Not applicable.

## Supplementary Materials

The following supporting information can be downloaded at: https://www.mdpi.com/article/10.3390/ani12192537/s1, Figure S1: Pedigree of the colonies used to generate germplasm and offspring; Table S1: Slicer software settings used for 3-D printed collection chamber; Table S2: Printer hardware features; Table S3: Number of eggs used and larvae produced from fertilization by use of sperm cryopreserved in various conditions.
