# Supplementary material for: Cryopreservation of Hydractinia symbiolongicarpus Sperm to Support Community-Based Repository Development for Preservation of Genetic Resources
Source: Animals (Basel). 2022 Sep 22;12(19):2537. doi: 10.3390/ani12192537 (PMC9559378; doi:10.3390/ani12192537)
Supplement: Supplementary file 1 [file animals-12-02537-s001.zip › Figure_S1.pdf]

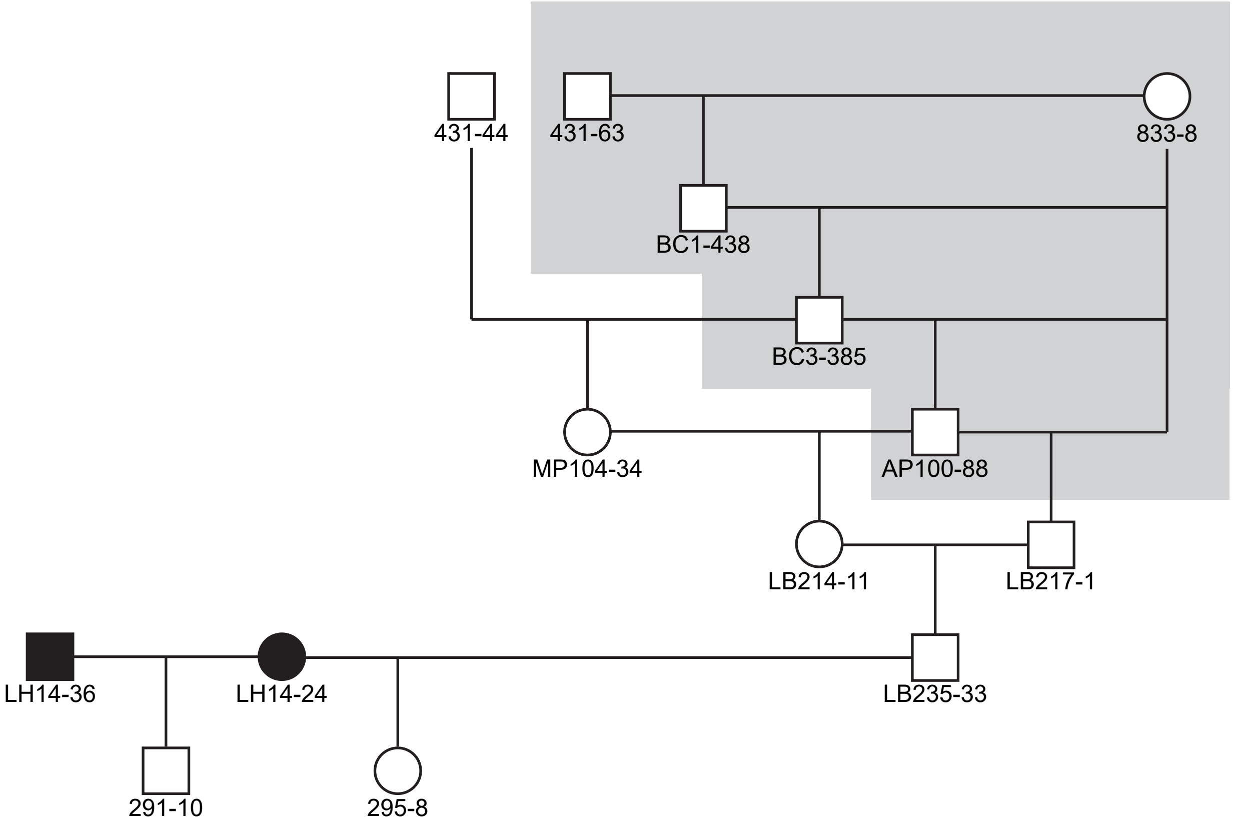

Supplemental Figure 1. Pedigree of the colonies used to generate germplasm and offspring.

Field-collected colonies are denoted with black symbols. Colony 291-10 is the offspring of two colonies collected from Lighthouse Point, New Haven, CT in 2014. Colony 295-8 is the offspring of a field collected colony and a laboratory strain, 235-33. The pedigree of colony 235-33 can be recreated by concatenating previously published pedigrees (shaded area) (Cadavid et al. 2004; Powell et al. 2007). Colony AP100-88 is from the mapping population in Powell et al. (2007). Colony 431-44 is from the mapping population in Cadavid et al. (2004).
