# Supplementary material for: Cryopreservation of Hydractinia symbiolongicarpus Sperm to Support Community-Based Repository Development for Preservation of Genetic Resources
Source: Animals (Basel). 2022 Sep 22;12(19):2537. doi: 10.3390/ani12192537 (PMC9559378; doi:10.3390/ani12192537)
Supplement: Supplementary file 1 [file animals-12-02537-s001.zip › Table_S1.pdf]

Table S1. Slicer software settings used to 3-D print collection chamber.

Settings	Expression
General settings	
Hotend temperature	200°C
Print speed	60 mm/s
Nozzle type	Brass
Nozzle diameter	0.4 mm
Extrusion/line width	0.45 mm
Nominal layer height	0.2 mm
Retraction distance	1.5 mm
Retraction speed	30 mm/s
Printer bed temperature	60 °C
Part cooling fan speed	75%
First layer settings	
Extrusion/line width	0.45 mm
Layer height	0.2 mm
Print speed	36 mm/s
Heat block temperature	205 °C
Part specific settings	
Infill	100%
Infill pattern	Rectangular
Wall/perimeter layers	2
Top layers	3
Bottom layers	3
Support placement	None
Support overhang angle	n/a
Support density	n/a
Build surface adhesion	Skirt
