# Supplementary material for: Cryopreservation of Hydractinia symbiolongicarpus Sperm to Support Community-Based Repository Development for Preservation of Genetic Resources
Source: Animals (Basel). 2022 Sep 22;12(19):2537. doi: 10.3390/ani12192537 (PMC9559378; doi:10.3390/ani12192537)
Supplement: Supplementary file 1 [file animals-12-02537-s001.zip › Table_S2.pdf]

Table S2. Printer hardware features.

Variable	Expression
Printer	Prusa i3 MK3
Power supply voltage	24 v
Extrusion	Direct drive
Filament size	1.75 mm
Filament supplier and type	PLA
Filament storage conditions	63-L plastic bin
Build surface size	21 x 21 cm
Build surface	PEI
Cooling fan	Blower
Cooling fan size	51.5 x 51.5 x 15 mm
Cooling fan voltage	5 v
Auto bed leveling sensor	PINDA proximity sensor
