# Supplementary material for: Cryopreservation of Hydractinia symbiolongicarpus Sperm to Support Community-Based Repository Development for Preservation of Genetic Resources
Source: Animals (Basel). 2022 Sep 22;12(19):2537. doi: 10.3390/ani12192537 (PMC9559378; doi:10.3390/ani12192537)
Supplement: Supplementary file 1 [file animals-12-02537-s001.zip › Table_S3.pdf]

Table S3. Number of eggs used and larvae produced from the fertilization by use of sperm cryopreserved in various conditions.

Cooling Rate	Cryoprotectant	Number of Eggs Used in IVF			Number of Larvae Produced		
		Replicate 1	Replicate 2	Replicate 3	Replicate 1	Replicate 2	Replicate 3
5 °C/min	5% DMSO	582	247	123	331	152	68
	10% DMSO	545	229	152	298	127	95
	15% DMSO	599	192	98	351	118	70
	5% Methanol	375	191	186	232	112	152
	10% Methanol	451	192	195	220	63	155
	15% Methanol	498	164	193	213	5	147
10 °C/min	5% DMSO	429	169	113	251	96	83
	10% DMSO	466	95	129	277	44	116
	15% DMSO	535	109	122	286	37	82
	5% Methanol	735	622	94	365	389	75
	10% Methanol	345	471	97	115	226	79
	15% Methanol	357	356	64	145	272	46
20 °C/min	5% DMSO	934	249	60	578	138	43
	10% DMSO	775	124	65	613	67	48
	15% DMSO	447	165	49	214	92	33
	5% Methanol	615	217	38	287	126	30
	10% Methanol	321	124	30	179	99	18
	15% Methanol	326	210	34	122	154	9
